# The Use of Flagella and Motility for Plant Colonization and Fitness by Different Strains of the Foodborne Pathogen *Listeria monocytogenes*

**DOI:** 10.1371/journal.pone.0005142

**Published:** 2009-04-09

**Authors:** Lisa Gorski, Jessica M. Duhé, Denise Flaherty

**Affiliations:** Produce Safety and Microbiology Research Unit, Agricultural Research Service, U.S. Department of Agriculture, Albany, California, United States of America; University of Wisconsin-Milwaukee, United States of America

## Abstract

The role of flagella and motility in the attachment of the foodborne pathogen *Listeria monocytogenes* to various surfaces is mixed with some systems requiring flagella for an interaction and others needing only motility for cells to get to the surface. In nature this bacterium is a saprophyte and contaminated produce is an avenue for infection. Previous studies have documented the ability of this organism to attach to and colonize plant tissue. Motility mutants were generated in three wild type strains of *L. monocytogenes* by deleting either *flaA*, the gene encoding flagellin, or *motAB*, genes encoding part of the flagellar motor, and tested for both the ability to colonize sprouts and for the fitness of that colonization. The *motAB* mutants were not affected in the colonization of alfalfa, radish, and broccoli sprouts; however, some of the *flaA* mutants showed reduced colonization ability. The best colonizing wild type strain was reduced in colonization on all three sprout types as a result of a *flaA* deletion. A mutant in another background was only affected on alfalfa. The third, a poor alfalfa colonizer was not affected in colonization ability by any of the deletions. Fitness of colonization was measured in experiments of competition between mixtures of mutant and parent strains on sprouts. Here the *flaA* and *motAB* mutants of the three strain backgrounds were impaired in fitness of colonization of alfalfa and radish sprouts, and one strain background showed reduced fitness of both mutant types on broccoli sprouts. Together these data indicate a role for flagella for some strains to physically colonize some plants, while the fitness of that colonization is positively affected by motility in almost all cases.

## Introduction

*Listeria monocytogenes* is a Gram-positive, saprophytic, soil bacterium that can become associated with food including fresh produce. When ingested, it can cause systemic foodborne illness in susceptible populations such as the elderly, the immunocompromised, pregnant women, and neonates with a fatality rate of approximately 25% [1]. While the lifestyle and physiology of *L. monocytogenes* as it relates to virulence is well studied [2], [3], less well understood is the physiology and molecular determinants important for interactions with plants. Surveys have detected *L. monocytogenes* on supermarket produce, including sprouts, and outbreaks have originated from contaminated produce and alfalfa tablets [4]–[7]. Previous work with the colonization of alfalfa sprouts showed that *L. monocytogenes* had strain-specific colonization of alfalfa that reflected differences in attachment of the cells to the sprouts [8]. Extension of that work into broccoli and radish sprouts showed that many *L. monocytogenes* strains were capable of colonizing all three sprout types to varying abilities, and that colonization was at least somewhat dependent on a strain's ability to withstand oxidative stress, but not acid stress [9].

Cell surface features such as pili, fimbriae, and flagella can be used by bacteria to attach to various surfaces [10], [11]. Pili and fimbriae have not been described for *L. monocytogenes*. In general flagella may be used directly for attachment and colonization as an adhesin, or indirectly to provide movement of the cell to the surface to be colonized. Depending on the surface *L. monocytogenes* uses flagella in both direct and indirect ways for attachment and colonization to different surfaces. Motility is necessary for *L. monocytogenes* host cell invasion in tissue culture, but flagella themselves are not used as adhesins in that environment [12], [13]. Motility, and not the presence of flagella contribute to colonization of the gastrointestinal tract in mice [13]. Also, motility and not flagella *per se* were shown to be important for biofilm formation on polyvinyl chloride, glass, and stainless steel surfaces [14], [15]. However, in a previous study flagella were shown to act as means of attachment for *L. monocytogenes* to stainless steel [16]. The conflicting data suggest that strain background, nutrient environment, and the type of surface may play a role in the use of flagella for attachment. Adhesin studies with *L. monocytogenes* and surfaces have included work in tissue culture, animal models, and *in vitro* surfaces.

Previous work in our lab indicates that flagellar motility is needed for *L. monocytogenes* to attach to fresh cut radishes, [17], but that study did not differentiate between motility or the presence of flagella. That work was done on cut plant tissue over the course of a few hours. Colonization of intact, growing plant tissue involves not only adherence to the plant surface by the bacteria, but also growth on that surface over time via substrates present in the plant. Not all plants would be expected to provide the same environment for colonizing bacteria. The aim of the present study was to determine via mutant analysis if flagella and/or motility played roles in the colonization of sprouts by *L. monocytogenes*. Sprouts provide a simple system for assaying colonization by bacteria since they grow within a few days, the plant type can be easily varied, and they provide root and leaf environments for attachment and colonization. Three wild type *L. monocytogenes* strains were selected for this study, and they were chosen based on their abilities to colonize alfalfa, radish, and broccoli sprouts at high, medium, and low levels [8], [9]. In the three strains, markerless deletion mutants were generated in *flaA*, which encodes flagellin, the structural subunit for flagella, and *motAB*, which encodes part of the flagella motor. Both types of mutants should be non-motile; however, the *motAB* mutant should contain intact, yet non-functional flagella, effectively divorcing the presence of flagella from the process of motility. Thus, if flagella were important for colonization, and motility less so, a *motAB* mutant might not be affected in colonization. All of these strains were screened for their ability to colonize alfalfa, radish, and broccoli sprouts as well as their fitness for that colonization.

## Results

### Construction of *flaA* and *motAB* deletion mutants

Sequence analysis of the deletion constructs confirmed that 805 bp (93%) of the *flaA* gene were removed with the first 7 nucleotides intact before the deletion, resulting in an out of frame mutation. The *flaA* gene is monocistronic [18]–[20]. For *motAB*, 658 bp were removed from the middle to the end of the *motA* gene, leaving the first 52 amino acids of *motA* intact, and resulting in a deletion of 83% of *motA*. This was an out of frame deletion that also overlapped the translational start site for *motB*. Any product made from this construct would be nonsense.

**Figure 1:**
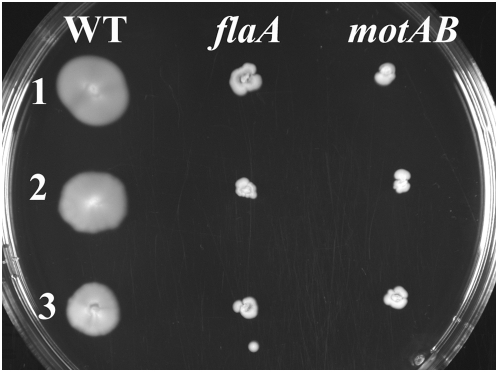
Colony spread of strains in soft agar. Strains were toothpicked onto BHI+0.4% agar, and grown at 30°C. Row 1 contains strains in the 10403 background, Row 2 contains strains in the RM2387 background, and Row 3 contains strains in the RM2992 background.

**Figure 2:**
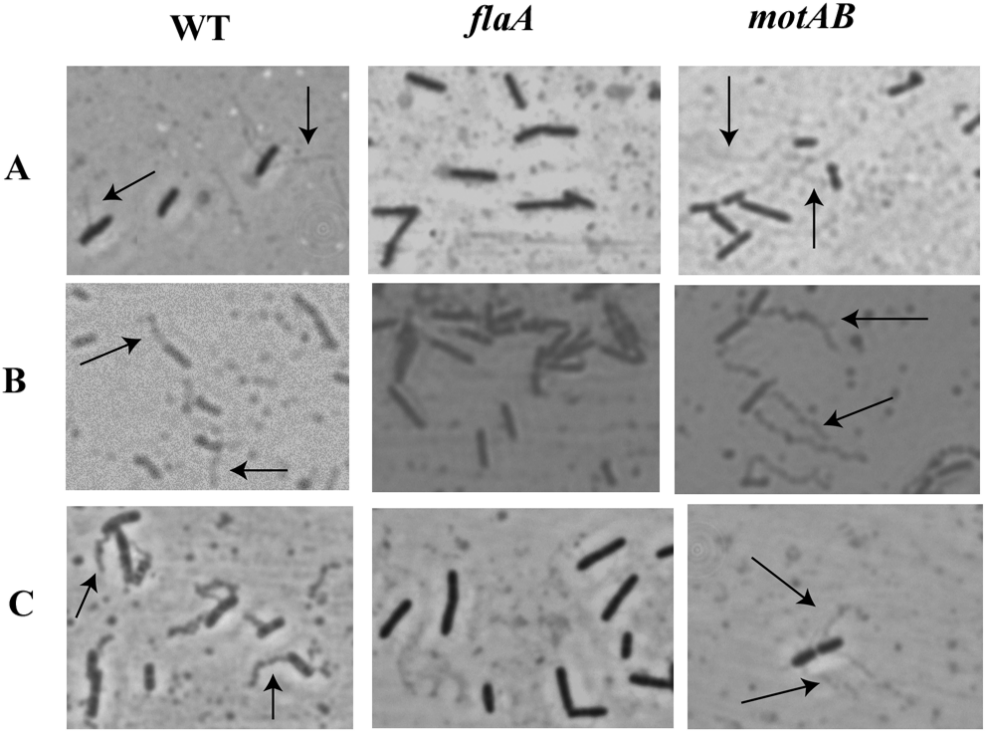
Flagellar stains of wild type and mutant strains. Some flagella are indicated by arrows. Shown are representative panels from the three different strain backgrounds: Row A) 10403 strain background, Row B) RM2387 strain background, Row C) RM2992 strain background. All micrographs were taken at 1000× magnification.

To determine if these mutants behaved as motility mutants, they were tested in soft agar to determine the amount of colony spread (Figure 1), and they were observed microscopically both in wet mounts and after flagellar staining (Figure 2). In motility agar all of the Δ*flaA* and Δ*motAB* mutants displayed small colony spreading phenotypes, indicating a lack of motility. Furthermore, all of the mutants were non-motile in wet mounts observed under the phase contrast microscope. Observations after flagellar staining showed that none of the Δ*flaA* mutants had visible flagella on their cell surfaces; whereas all of the wild types and the Δ*motAB* mutant strains did. These data indicate that the Δ*flaA* mutants were non-motile due to a lack of flagella, and the Δ*motAB* mutants were non-motile due to non-functional, but present flagella. Complementation of all the Δ*flaA* and Δ*motAB* mutants with a plasmid carrying the wild type gene in trans resulted in motile phenotypes (data not shown).

### Sprout colonization

Seeds were inoculated at Day 0 by exposure to a suspension of *L. monocytogenes* in water for 1 h after which the inoculum was removed. Sampling of the sprouts began at Day 1, and results shown for Days 1 and 3 (Table 1) reflect the number of CFU that grew on and attached to the sprouts from that initial seed inoculation. The broad range of alfalfa colonization that was previously reported for these three wild type strains [8] was confirmed with RM2992 being a poor colonizer, 10403 a mid-level colonizer, and RM2387 a very good colonizer reaching levels of 5 log CFU/sprout. Furthermore the levels of colonization for the wild type strains on radish and broccoli also agreed with previous experiments [9].

**Table 1:** Number (log CFU/sprout) of the different *L. monocytogenes* on sprouts after 1 and 3 days of growth.

Strain	Alfalfa		Radish		Broccoli	
	Day 1	Day 3	Day 1	Day 3	Day 1	Day 3
10403 wild type	0.6±0.6 A^a^	2.5±1.5 A	4.0±0.8 A	5.5±0.5 A	2.0±1.1 A	4.5±0.3 A
10403Δ*flaA*	0.3±0.3 A	1.0±0.8 B	3.9±0.9 A	5.4±0.2 A	1.8±1.3 A	4.7±0.6 A
10403Δ*motAB*	0.6±0.6 A	2.8±0.7 A	3.7±1.1 A	5.0±0.4 A	2.2±1.1 A	4.5±0.2 A
RM2387 wild type	1.4±0.9 A	5.1±0.4 A	4.7±0.7 A	6.7±0.3 A	2.5±0.6 A	5.1±0.5 A
RM2387Δ*flaA*	0.07±0.07 A	2.4±1.0 B	3.5±1.0 B	5.6±0.2 B	1.1±0.7 B	4.6±0.2 A
RM2387Δ*motAB*	0.25±0.25 A	4.0±1.1 A	4.7±0.8 A	6.7±0.5 A	2.6±0.6 A	5.1±0.4 A
RM2992 wild type	0.2±0.2 A	0.6±1.0 A	3.9±0.8 A	5.3±0.3 A	1.1±1.0 A	3.9±0.6 AB
RM2992Δ*flaA*	0.2±0.2 A	0.7±0.5 A	3.7±0.7 A	5.2±0.4 A	0.6±0.5 A	3.1±0.6 A
RM2992Δ*motAB*	0.2±0.2 A	1.1±1.1 A	3.6±0.5 A	5.6±0.3 A	0.9±0.8 A	4.3±0.6 B

a For each set of parent and isogenic mutant strains within a column, numbers followed by the same letter are not statistically different, and a different letter indicates a statistical difference.

As for the motility mutants, none of the Δ*motAB* mutants showed any difference in colonization from their parent strains when tested in single culture. However, some of the Δ*flaA* mutants were reduced in colonization on some sprout types. Strain 10403Δ*flaA* was reduced in colonization by Day 3 by over 1 log CFU/sprout from the 10403 parent (P<0.01) only on alfalfa. RM2387Δ*flaA* was reduced in sprout colonization on all the sprout types when compared with its parent. It had a 3 log reduction by Day 3 on alfalfa (P<0.0001), a one log reduction on Days 1 and 3 on radish (P<0.01), and over a one log reduction on broccoli on Day 1 (P<0.01), which was overcome by Day 3 when it was equivalent to the parent strain. For strain RM2992 there were no significant differences for any of the mutant strains from their wild type parent strains.

The Δ*flaA* strains that had phenotypes on sprouts were complemented with copies of their parent *flaA* genes in trans (Table 2). Complementation experiments in this system were somewhat problematic. Antibiotics added to the sprouting system led to wildly variable colonization levels, so the experiments were run without antibiotic selection. There is some plasmid loss without antibiotic selection, so colonization levels similar to the parent strains would not be expected due to variable expression of the complement plasmid. Expression of *flaA* in trans in both 10403Δ*flaA* and RM2387Δ*flaA* complemented some defects. Specifically 10403Δ*flaA* on alfalfa was complemented by Day 3 (P<0.0001), as was RM2387Δ*flaA* on alfalfa and radish by Day 3 (P<0.05) in comparison to the vector control strains. The colonization defects of RM2387Δ*flaA* on alfalfa and radish on Day 1 were not complemented, but the vector controls showed higher values than their non-plasmid carrying counterparts. However the RM2387Δ*flaA* complemented strain on broccoli was rescued on Day 1 (P<0.02). The *flaA* gene from RM2387 was placed into strains 10403Δ*flaA* and RM2992Δ*flaA* to see if they enhanced colonization by those mutant strains, and no enhancement was seen (data not shown). None of the mutants or their complements had any growth kinetic defects in complex media when compared to their control strains.

**Table 2:** Complementation of affected *flaA* mutants on sprouts.

Strain	Alfalfa		Radish		Broccoli	
	Day 1	Day 3	Day 1	Day 3	Day 1	Day 3
10403Δ*flaA*+vector	2.0±0.6 A^b^	2.7±0.1 A	ND^a^	ND	ND	ND
10403Δ*flaA*+*flaA*	2.0±1.0 A	3.2±0.1 B	ND	ND	ND	ND
RM2387Δ*flaA*+vector	1.7±1.0 A	4.2±0.1 A	4.5±0.1 A	5.6±0.3 A	1.5±0.7 A	4.7±0.2 A
RM2387Δ*flaA*+*flaA*	0.8±0.8 A	5.0±0.1 B	4.5±0.1 A	6.3±0.3 B	2.3±0.1 B	4.6±0.6 A

Number (log CFU/sprout) on sprouts after 1 and 3 days of growth.

a Not Done (because the *flaA* mutants had no phenotype on sprouts).

b For each set of parent and isogenic mutant strains within a column, numbers followed by the same letter are not statistically different, and a different letter indicates a statistical difference.

### Fitness of sprout colonization

Mutant strains that displayed no statistical difference from their parent strains when tested for sprout colonization in single culture were tested in 1∶1 mixtures with the parent strains to determine if there were any differences in fitness of colonization. The RM2992 strains were not tested on alfalfa due to the very low numbers of colonies that result. Table 3 presents the percent of each isogenic pairing that were non-motile after three days of growth with sprouts. A result of 50% indicates equal fitness between the parent and mutant strains. In all cases tested, the Δ*flaA* mutants competed poorly with the wild type strains, ranging from 4% recovery for 10403Δ*flaA* on radish sprouts to 11.5% recovery for RM2387Δ*flaA* on broccoli sprouts. The Δ*motAB* mutants fared better in competition than did the Δ*flaA*:wild type pairings, but several of the Δ*motAB* strains displayed reduced fitness on sprouts including 10403Δ*motAB* on all sprout types, RM2387Δ*motAB* on alfalfa and radish, and RM2992Δ*motAB* on radish sprouts. However, both RM287Δ*motAB* and RM2992Δ*motAB* were as fit as their parent strains on broccoli sprouts. In the five instances where fitness was measured for both isogenic Δ*flaA* and Δ*motAB* mutants, there were statistical differences between the non-motile strains in fitness only for the RM2387 and RM2992 backgrounds on broccoli with the Δ*flaA* mutants reduced in comparison with the Δ*motAB* mutants by a factor of five.

**Table 3:** Percent Non-motile resulting from 1∶1 Competition between Wild Type and Mutant Strains on Sprouts after 3 days of Colonization.

Strain	Alfalfa	Radish	Broccoli
10403 : Δ*flaA*	ND^a^	4.5±4.0 A^b^	10.0±6.7 A
10403 : Δ*motAB*	36.6±12.5	14.9±8.3 A	18.8±8.1 A
RM2387 : Δ*flaA*	ND	ND	11.5±5.2 A
RM2387 : Δ*motAB*	31.5±9.1	26.4±8.2	54.9±14.1 B
RM2992 : Δ*flaA*	ND	8.3±5.1 A	10.5±5.6 A
RM2992 : Δ*motAB*	ND	19.0±5.9 A	44.8±14.4 B

a Not Done.

b For each set of parent and isogenic mutant strains within a column, numbers followed by the same letter are not statistically different, and a different letter indicates a statistical difference.

### Sequence analysis of *flaA* alleles

The *flaA* alleles of the three wild type strains were amplified by PCR and sequenced to determine if differences in the single culture phenotypes of the different *flaA* mutants on sprouts could be explained by sequence differences in the alleles. Two independent PCR reactions were done for each *flaA* gene, and the duplicates were sequenced to ensure that no mutations were introduced during PCR. For all three the coding regions were 864 nucleotides long, which translates into a protein of 287 amino acids, and agrees with all other published sequence lengths of *flaA* from *L. monocytogenes*. The sequences of *flaA* in RM2387 and RM2992 were identical at both the DNA and protein level. The *flaA* gene from these two serotype 4b strains was also identical to the serotype 4b outbreak strain F2365 [20]. The DNA sequence of *flaA* for strain 10403 had 15 individual nucleotide changes in comparison with the others, but these changes translate into a one amino acid difference with a glutamine instead of a glutamate predicted at amino acid position 198.

## Discussion

The three wild type *L. monocytogenes* strains were selected because of their demonstrated differing abilities to colonize alfalfa, radish, and broccoli sprouts [9]. Previous work indicated that motility played some role in plant attachment [17], but it was not clear whether that attachment was due to the presence of flagella or to the process of motility itself. In the present study the only mutants to display a colonization phenotype in single culture were Δ*flaA* mutants. This would seem to indicate that the presence of flagella and not motility is important for the colonization of some plant tissues by some *L. monocytogenes*. Additionally, fitness studies with wild type and isogenic mutants in co-culture with sprouts indicated that motility itself contributed toward fitness of colonization in some cases.

Flagella may play a more significant role in the colonization of alfalfa than the other plants since both strains capable of alfalfa colonization (10403 and RM2387) showed reductions in single strain colonization when there was no *flaA* expression (Table 1). Furthermore while the loss of *flaA* led to a reduction of colonization in the RM2387 background for all of the sprouts, the defect was most pronounced on alfalfa with a 3 log reduction compared to 1–2 log reductions for radish and broccoli. Of the three wild type strains, RM2387 was the most robust colonizer and only RM2387 showed defects with a *flaA* deletion on radish and broccoli sprouts. It is possible that the flagella of RM2387 possess traits that allow for better attachment and/or colonization to the plant surface. The deletion of *flaA* did not obliterate colonization for any strain, so while flagella may be necessary for some strains to colonize some plants, they are not the only bacterial factor playing a role in the interaction. We added the *flaA* gene from RM2387 to both 10403Δ*flaA* and RM2992Δ*flaA* to see if colonization ability would be enhanced in the latter two strains, but it was not, suggesting that it is not the product of the *flaA* gene itself that helps to determine colonization, but possibly additional factors, including some that may be decorating the flagella. The fact that there were no sequence differences between the *flaA* genes of RM2387 and RM2992 is further support of this.

Other explanations for the high colonization ability of RM2387 are the potential presence of additional colonization factors not present in the other strains, and that its flagellar complement may be larger than the others. Also the finding that the RM2992 wild type was equivalent to both its Δ*flaA* and Δ*motAB* mutants for sprout colonization could indicate that RM2992 does not make flagella in this system. Attempts to assess expression of *flaA* in the sprout system were met with great technical difficulties due to the tightness of the interactions of the *L. monocytogenes* cells to the plant tissue and, with RM2992, very few cells present on the plant tissue so that RNA could not be isolated. However, the finding that the non-motile RM2992 mutants did show phenotypes in the experiments measuring fitness of colonization (Table 3) implies that this strain did produce flagella in the system.

While motility itself was not needed for colonization in single culture the data from the competition experiments showed that it contributed to the fitness of colonization (Table 3). Fitness was reduced in almost all the Δ*motAB* strains, and these reductions were statistically similar to the reductions in fitness by isogenic Δ*flaA* mutants in most cases when both were tested. Fitness was not assessed in mutants that displayed a colonization defect in single culture as those Δ*flaA* mutants were obviously not competitive with the wild type. Fitness for colonization of alfalfa and radish sprouts by all three strains required motility for full efficiency; however, full motility played a role in fitness for broccoli colonization only in the 10403 strain background. RM2387Δ*motAB* and RM2992Δ*motAB* had wild type fitness on broccoli.

Previous studies demonstrate that *L. monocytogenes* preferentially colonizes the roots of these sprouts [8], [9]. The literature regarding the use of flagella for attachment to plant roots is mixed, and can vary among different plants and bacteria. In the colonization of various plants by *Pseudomonas* spp., flagella, motility, and chemotaxis are important some for plant attachment and/or colonization [21], and non-motile mutants were defective in the colonization of tomato and potato roots, [22]–[24]. Martínez-Granero *et al* [25] showed that highly motile variants of *P. fluorescens* were enhanced in the ability to colonize alfalfa roots over strains with normal motility, but this study did not address if it was motility or the presence of flagella that were important for the enhanced colonization. However in other studies some motility mutants of *Pseudomonas* species, were not defective in root colonization of wheat and soybean [26], [27], Flagella are important for colonization and attachment of *Azospirillum brasilense* to wheat roots [28]. Root exudates from lettuce induce *Salmonella enterica* to chemotax toward the roots, and is thought to positively influence plant colonization efficiency [29]. Flagella-minus, non-motile mutants of *Salmonella enterica* Senftenberg were defective in attachment to basil leaves [30].

Since RM2387 and RM2992 had identical sequences, the difference in single culture sprout colonization by these two serotype 4b strains cannot be explained by the sequence of the flagellin protein. However, the flagellin protein in *L. monocytogenes* is post-translationally modified by glycosylation [31]. It is possible that the differences in colonization seen by the three wild type strains, and the differential contribution of flagella to that colonization among the strains is caused by chemical modifications on the flagella structures. The bacterial strains may also have differential expression of other adhesin factors that are not related to motility. Plant lectins may also contribute to the attachment and colonization process, as it does in other bacteria such as *Rhizobium* [32], and *L. monocytogenes* does react to different plant lectins in a strain-specific fashion [33], [34]. Plant factors and attachment sites may also account for fitness differences seen among the strains, especially with broccoli sprouts.

The role of flagella and motility in the *L. monocytogenes*-plant system differs from that seen in the *L. monocytogenes* culture with animal cells where motility and not flagella is important for invasion of tissue culture cells. *L. monocytogenes* Δ*flaA* mutants are defective in the invasion of tissue culture cells but not in cell-to-cell spread [12]. O'Neil and Marquis [13] demonstrated that the defect in tissue culture cell invasion was due to a lack of motility and not a lack of flagella, because a *motB* mutant was as deficient in invasion as a Δ*flaA* mutant. This means that flagella do not act as attachment factors for tissue culture cells. Similarly motility, but not flagella *per se* were shown to be important for biofilm formation by *L. monocytogenes* in both static and flow cell biofilm systems [14], [15]. However, flagella do play a role in the attachment of *L. monocytogenes* to stainless steel [16]. In the colonization of plants, the role of motility and flagella may be more complicated. The contributions of flagella and motility by *L. monocytogenes* to colonization were dependent upon the strain and the plant. In general, motility played a role in fitness for sprout colonization. In some strains the presence of flagella itself was a determinant for colonization, indicating a direct role for flagella in part of the colonization process for some strains on some plants. It is likely that contributions from chemotaxis and attachment via flagella are needed in some systems for full colonization ability by *L. monocytogenes*, and further study of plant attractants and attachment sites is required to determine the exact role of each in the system.

## Materials and Methods

### *L. monocytogenes* strains, plasmids, media, and culture conditions

The strains used in this study are listed in Table 4. Deletion mutants of *flaA* and *motAB* were constructed in three *L. monocytogenes* wild type strain backgrounds. Routinely, strains were grown in TSYE medium (Tryptic Soy Broth without dextrose+0.6% yeast extract, Difco, Becton Dickinson, Franklin Lakes, NJ) or Brain Heart Infusion Medium (BHI, Difco) at 30°C. Modified Oxford Agar (MOX, Difco, Becton Dickinson, Franklin Lakes, NJ), which inhibits most Gram-negative bacteria, as well as many Gram-positives, was used to selectively grow *L. monocytogenes* from inoculated seeds/sprouts. *Escherichia coli* strains were grown on Luria Broth or Luria Agar (LB, Difco). Deletion constructs were made in pAUL-A [35], which was maintained in *Escherichia coli* with erythromycin (300 µg/ml), and in *L. monocytogenes* with erythromycin (1 µg/ml) and lincomycin (25 µg/ml). Complementation plasmids were constructed in pAT18 [36], which was maintained in both *E. coli* and *L. monocytogenes* with the same antibiotics used for pAUL-A. Phosphate buffered saline (PBS) contained 150 mM NaCl and 10 mM sodium phosphate (pH 7.2).

**Table 4:** Strains used in this study.

Strain	Description	Source or reference
*L. monocytogenes*		
10403	Serotype 1/2a, animal isolate	D. Portnoy, UC Berkeley
RM5708	10403, Δ*flaA*	This study
RM4493	10403, Δ*motAB*	This study
RM2387	Serotype 4b, mint isolate	[17]
RM5345	RM2387, Δ*flaA*	This study
RM4494	RM2387, Δ*motAB*	This study
RM2992	Serotype 4b, cucumber isoate	M. Borucki, USDA, ARS
RM5346	RM2992, Δ*flaA*	This study
RM4720	RM2992, Δ*motAB*	This study
*E. coli*		
DH10B	Cloning Strain	

### Construction of mutants

The *flaA* and *motAB* genes were cloned from each of the three wild type strains' chromosomes using primers that were designed against the *L. monocytogenes* EGD-e genome sequence [19]. The primer sequences are in Table 5. BamHI or KpnI restriction sites were engineered into the primers to facilitate cloning. PCR was performed with the Expand High Fidelity PCR kit (Roche, Indianapolis, IN) and cloned into the vector pDrive using the Qiagen PCR cloning kit (Qiagen, Inc., Valencia, CA) or the vetor pCR2.1 using the Invitrogen TOPO TA cloning kit (Invitrogen Corp., Carlsbad, CA). The genome regions were then subcloned into pAUL-A, a shuttle vector used for allelic exchange in *L. monocytogenes* [35]. Markerless deletion mutants were constructed in the *flaA* and *motAB* genes using inverse PCR with primers that had restriction sites engineered into the 5′ ends. The central portion of the genes were deleted using inverse PCR with the Expand Long Template PCR kit (Roche) using internal primers in which restriction sites were engineered into the 5′ ends. The resulting amplicons were cut with the restriction enzymes in the deletion primers, self-ligated, electroporated into *E. coli* DH10B, and selected on LB+erythromycin agar. The sequence of the deletion constructs was confirmed by sequencing on an ABI Prism 3130×l DNA Analyzer, using Big Dye Terminator v. 3.1 chemistry (Applied Biosystems, Foster City, CA). The deletion plasmids were electroporated into each of the three wild type strains of *L. monocytogenes* [8] and selected on BHI agar with erythromycin and lincomycin. Allelic exchange was done as described except that erythromycin and lincomycin were used instead of just erythromycin [37]. The physical structure of the chromosomal deletions in the mutants was confirmed by PCR using the primers used to originally amplify the genome regions from the *L. monocytogenes* wild type strains, and comparing the sizes of the products to the wild type clones and the deletion constructs.

**Table 5:** PCR primers used in this study.

Gene and purpose	Primer Name	Primer sequence (5′→3′)	Restriction site^a^
*flaA* cloning	flaA PCR for	GCGGATCCGCAACGATCCGCAATGTCTTCC	BamHI
	flaA PCR rev	GCGGATCCACTTCCGTATCTGCGCCTTCAATCACTAAA	
*flaA* deletion	fla002	AAAGCTCGAGCTTCTCAAGCAAACCAAACACC	XhoI
	fla400	AAAGCTCGAGCTTTCATTTGTGTTTCCCTCCTAC	
*motAB* cloning	cheR K for	TGGGTACCCAAGCTATATTACGAACCGCGAC	KpnI
	che R K rev	CTGGTACCGGATAAAAACGGCTCTGGCAAAC	
*motAB* deletion	mot002	ATAGATCTGGGTGCGCCATCATAACAGC	BglII
	mot101	CGAGATCTGCCAAGCGTCGCAAGAAACC	

a Restriction site in primer is underlined.

Complementation plasmids were constructed in pAT18 [36] using the cloned *flaA* genes from both 10403 and RM2387, and the *motAB* operon from 10403.

### Flagellar staining and assessment of motility

Motility was assessed microscopically in wet mounts on cultures grown overnight at 30°C in BHI broth, and by monitoring colony spread on BHI+0.4% agar plates grown overnight at 30°C. Flagellar staining was done using Becton-Dickinson flagella stain (Becton-Dickinson), following the manufacturer's instructions with the exception that the cultures were grown on trypticase soy agar with 5% sheep blood at 30°C.

### DNA Sequence Analysis

The *flaA* genes from all three wild type strains were PCR amplified with the Expand High Fidelity PCR kit (Roche), sequenced, and compared. DNA sequences were assembled and analyzed with DNA Star (DNA Star, Madison, WI). Two separate PCR reactions were done for each *flaA* allele, and were sequenced independently to ensure that mutations were not introduced during PCR. The GenBank accession numbers for the three *flaA* sequences are FJ234181 for 10403 *flaA*, FJ234182 for RM2387 *flaA*, and FJ234183 for RM2992 *flaA*.

### Assay for growth of *L. monocytogenes* on sprouts

Alfalfa seeds were purchased from International Specialty Supply (Cookeville, TN), and radish and broccoli seeds were purchased from The Sproutpeople (Poulsboro, WA). Seeds were sanitized with Ca(OCl)_2_ as described, and washed and soaked for 4 h in 100×15 mm Petri dishes containing sterile water to remove the disinfectant 8, 9. After soaking, alfalfa, radish, and broccoli seeds were inoculated with strains of *L. monocytogenes* as previously described [8]. Briefly, 20 ml of an aqueous cell suspension of *L. monocytogenes* (10^4^ CFU/ml) was added to the seeds in Petri dishes, and incubated at room temperature on a rotating shaker (The Belly Dancer, Stovall Life Science, Greenboro, NC) for 1 h. The cell suspension was removed and replaced with sterile water. The seeds were then incubated and sprouted at room temperature on the rotating shaker for three days. Each day, the irrigation water was removed and replaced with sterile water. Sprout sampling was done within 30 minutes of these water changes. At these time points, sprouts were placed into 300 µl of PBS, and homogenized using a sample pestle (Scienceware™, BelArt #F19922-0001) attached to a cordless rotary tool (Dremel, Racine, WI) set at 7500 rpm for 2–3 seconds. The suspension was dilution plated onto MOX agar, and the plates incubated at 30°C for two days. *L. monocytogenes* colonies are characteristically bluish-white on this selective and differential medium, which inhibits the growth of most Gram-negative bacteria.

### Competition experiments

Mutants displaying no phenotype when tested for sprout colonization in single culture were tested in competition with the wild type after three days of sprout colonization to assess fitness of colonization. Strains were mixed in a 1∶1 combination before inoculation of seeds as described above. Inoculating suspensions were dilution plated onto BHI agar to confirm that the ratio was 1∶1. Each experiment used three separate dishes of each sprout. Irrigation water was changed daily, and three sprouts were sampled per dish, crushed, and dilution plated on MOX on Day 3. MOX plates were incubated, and 96 colonies from each sprout plating were picked at random and transferred individually with a sterile toothpick into a well of a 96 well microtitre plate that contained BHI+0.4% agar. These microtitre plates were incubated at 30°C overnight. The numbers of motile and non-motile colonies were counted and the percentage non-motile of the total was calculated.

### Statistics

All sprout colonization experiments were conducted at least four times with at least three replicates and two separate cultures for each. Data is presented as the average of all experiments with standard deviation. GraphPad Prism version 5.01 (GraphPad Software, San Diego, CA) was used for statistical analysis. Two Way ANOVA with Bonferroni post tests or unpaired *t*-tests with Welch correction were performed on the sprout colonization data to compare the results for the mutant strains with their wild type parent strains and complemented strains to vector control strains to determine statistical differences. Statistical differences in fitness between isogenic Δ*flaA* and Δ*motAB* strains were determined by the Kruskal-Wallis test with Dunn's Multiple Comparison post-test.

GenBank Accession Numbers for sequences presented in this paper: FJ234181, FJ234182, FJ234183
